# Dietary Choline Supplementation Modulates Growth Performance and Protein Metabolism by Promoting Glucose and Lipid Catabolism in Chinese Perch (*Siniperca chuatsi*)

**DOI:** 10.3390/ani15131926

**Published:** 2025-06-30

**Authors:** Wanjia Zhu, Yi Yi, Liwei Liu, Zhiwei Zou, Jianming Chen, Jianmei Su

**Affiliations:** 1 Hubei Key Laboratory of Regional Development and Environmental Response, Faculty of Resources and Environmental Science, Hubei University, Wuhan 430062, China; 202321108012137@stu.hubu.edu.cn (W.Z.); 202421108012111@stu.hubu.edu.cn (Z.Z.); 2 College of Fisheries, Chinese Perch Research Center, Engineering Research Center of Green Development for Conventional Aquatic Biological Industry in the Yangtze River Economic Belt, Ministry of Education, Huazhong Agricultural University, No. 1, Shizishan Street, Hongshan District, Wuhan 430070, China; HZNYDXYY@163.com (Y.Y.); liuliwei@mail.hzau.edu.cn (L.L.); 3 Key Laboratory of Healthy Freshwater Aquaculture, Ministry of Agriculture and Rural Affairs, Key Laboratory of Fish Health and Nutrition of Zhejiang Province, Zhejiang Institute of Freshwater Fisheries, Huzhou 313001, China

**Keywords:** *Siniperca chuatsi*, choline, feeding, glucose and lipid metabolism, protein utilization, growth

## Abstract

An 8-week trial was conducted to investigate the effects of choline on the growth of Chinese perch (85.57 ± 0.54 g) with dietary choline supplementation at 0 (P0), 400 (P1), 800 (P2), 1600 (P3), 3200 (P4), and 6400 mg/kg (P5). Protein efficiency ratio and protein retention value (PRV) were higher in the P1–P4 groups than in the P0 group (*p* < 0.05). Compared to the P0 group, weight gain rate and specific growth rate (SGR) increased in the P2 and P3 groups (*p* < 0.05). The orexigenic gene *agrp* expression level rose in the P2–P4 groups (*p* < 0.05). The expression level of the lipolysis-related gene *hsl* or *pparα* was elevated in the P2 and P4 groups (*p* < 0.05). Proteolysis-related gene (*ampd*, *mafbx*, and *murf1*) expressions decreased in the P1–P4 groups, while *tor* and *gk* gene expressions increased in the P2 and P3 groups (*p* < 0.05). Broken-line analysis indicated that the optimal choline supplementation level for Chinese perch is 788.38 mg/kg based on SGR and 851.04 mg/kg based on PRV. The results demonstrate that moderate dietary choline supplementation enhances growth performance by promoting glucose and lipid catabolism and inhibiting protein catabolism in Chinese perch.

## Simple Summary

Choline plays pivotal roles in growth, nutrient digestion, and energy metabolism for fish health. This study systematically investigated the effects of dietary choline supplementation on feeding behavior, growth performance, and nutrient and energy metabolism in Chinese perch. Our findings demonstrated that choline supplementation significantly elevated the expression level of the orexigenic gene *agrp*, thereby enhancing the feed intake of fish. Furthermore, choline supplementation activated the expression of glycolysis-related genes (*pk* and *gk*), lipolysis-related genes (*hsl* and *pparα*), and the energy metabolism-related gene *tor* while suppressing the expression of proteolysis-related genes (*ampd*, *mafbx*, and *murf1*). The results demonstrate that dietary choline supplementation enhances the growth performance of fish by promoting glucose and lipid catabolism and inhibiting protein catabolism. These findings provide critical insights into choline-modulated optimization of energy utilization, emphasizing the necessity of moderate choline supplementation for the ideal growth of Chinese perch.

## 1. Introduction

Chinese perch (*Siniperca chuatsi*) has unique feeding characteristics. It feeds exclusively on live prey and requires artificial training to accept formulated feeds [1,2]. This fish has exceptionally high protein requirements, with premium protein sources (primarily fishmeal) accounting for the majority of the cost of its diet [3]. Developing formulated feeds and alternative protein sources are imperative for sustainable aquaculture practices [4]. However, studies have revealed that substituting plant protein for fish meal not only compromises protein digestibility and utilization efficiency in Chinese perch but also leads to immunosuppression, growth retardation, and nutritional fatty liver disease in fish, which is primarily attributed to the poor palatability and amino acid imbalance in plant-based diets [5,6,7]. These findings highlight the urgent need to develop functional feed additives with appetite-stimulating and digestive-enhancing properties to improve the growth performance and feed utilization efficiency of Chinese perch [8].

Choline, with the molecular formula (CH_3_)_3_N(CH_2_)_2_OH), is a potent organic alkali and essential water-soluble vitamin-like substance for fish survival and growth [9,10]. Due to the low enzymatic activity for endogenous choline synthesis in fish, autogenous choline production cannot meet the demands of rapid growth, so dietary choline supplementation is necessary to fulfill the physiological requirements of fish [11]. Choline can participate in the synthesis and metabolism of phospholipids, amino acids, and nucleotides by donating methyl groups, which are deficient in fish [9]. Dietary choline can be converted into betaine, which facilitates methionine synthesis, thereby enhancing the availability of essential amino acids, promoting protein deposition, and improving feed utilization efficiency [12]. A previous study has demonstrated that dietary choline supplementation at 770–1082 mg/kg significantly increased the feed intake, weight gain rate, and feed efficiency of grass carp (*Ctenopharyngodon idella*), as well as elevating its muscle protein, lipid, and amino acid content [13].

Choline is the biosynthetic precursor of the neurotransmitter acetylcholine. Dietary choline supplementation can enhance the synthesis of acetylcholine, stimulate acetylcholine receptors, elevate cerebral acetylcholine levels, activate the feeding center, and increase the feed intake of fish, thereby improving their energy provision and growth [14,15,16,17]. Consequently, choline has been widely used as a feed additive and feeding stimulant in aquaculture to accelerate the growth rates of fish [18,19]. Furthermore, choline-derived phospholipids can not only maintain cellular membrane integrity but also regulate glucose and lipid metabolism in fish [8]. Specifically, in Chinese perch, phosphatidylcholine supplementation significantly up-regulated the expression of hepatic glucose metabolism-related genes (*glut2*, *gk*, *cs*, and *fas*) and enhanced glucose transport and oxidation, thereby reducing glucose levels in blood [20]. Baldissera et al. demonstrated that dietary choline supplementation at 800–1200 mg/kg increased hepatic pyruvate kinase activity and promoted ATP production to meet the energetic demands of the rapid growth of Nile tilapia (*Oreochromis niloticus*) [21]. Extensive research has confirmed that choline supplementation in fish feed promoted lipid catabolism, reduced lipid deposition, and prevented nutritional fatty liver formation; thus, choline is considered a vital anti-fatty liver factor [9,22,23]. For instance, dietary supplementation of 603–1512 mg/kg choline significantly reduced the hepatic lipid content in orange-spotted grouper (*Epinephelus coioides*) [24]. Conversely, choline deficiency induced multiple undesirable disorders in fish, including appetite suppression, growth retardation, and hepatic lipid dysregulation, as reported in grass carp, yellow catfish (*Pelteobagrus fulvidraco*), and pacu (*Piaractus mesopotamicus*) [25,26,27].

Research has demonstrated that the beneficial effects of choline on fish growth and metabolism are concentration-dependent and influenced by factors such as feed composition, species specificity, and developmental stages [19,28]. Dietary supplementation with choline at 15,000 mg/kg has been shown to alleviate high-lipid-diet-induced oxidative stress and hepatic damage in the hybrid grouper (♀*Epinephelus fuscoguttatus* × ♂*E. lanceolatu*) [19]. However, 20,000 mg/kg of choline supplementation significantly reduced the growth performance and survival rates of the hybrid grouper, suggesting the potential toxicity of choline at elevated concentrations [19]. Therefore, determining the optimal level of choline supplementation in feed is essential to enhance fish growth performance while reducing feed costs and avoiding the toxic effects of excessive choline supplementation. Although the effects of choline have been well documented in various fish species, its application in Chinese perch has received comparatively less attention. In this study, we conducted a 56-day feeding trial in which Chinese perch were fed diets containing graded choline supplementation levels (0–6400 mg/kg). This study systematically studied the effects of dietary choline supplementation on the growth performance, feeding behavior, glucose and lipid metabolism, protein utilization, and energy allocation patterns of Chinese perch, aiming to lay a theoretical foundation for optimizing the supplementation of choline in aquatic feed.

## 2. Materials and Methods

### 2.1. Preparation of Experimental Diets

Six isonitrogenous and isoenergetic experimental diets (crude protein: 46.88%; crude lipid: 11.88%; carbohydrate: 7.98%) were formulated for Chinese perch using fish meal and casein as the protein sources, with fish oil and soybean oil as the lipid source. Choline chloride (50% purity) was added to create six dietary choline supplementation concentrations: 0.0 mg/kg (P0), 400 mg/kg (P1), 800 mg/kg (P2), 1600 mg/kg (P3), 3200 mg/kg (P4), and 6400 mg/kg (P5). Each diet was assigned to a group of Chinese perch, with three replicates per group. The P0 group was set as the control.

The complete formulation and proximate composition of the diets are detailed in Table 1. Feed ingredients were sequentially blended in ascending order of inclusion levels to ensure homogeneity. Specifically, 20% casein was added to meet the protein and amino acid requirements of Chinese perch. Additionally, 4% fish oil and 4% soybean oil were premixed, followed by the mechanical dispersion of lipid microgranules into the powdered matrix. The mixture was pelletized (5 mm) using a feed extruder (Zhengchang Feed Machinery Co., Ltd., Liyang, China). All diets were vacuum-packed and stored at −20 °C during the trial.

### 2.2. Fish Husbandry

Chinese perch were obtained from the Xianning Haihui Aquaculture Cooperative (Xianning, China) and were acclimatized for 14 days at the Aquaculture Research Facility of Huazhong Agricultural University (Wuhan, China). During acclimation, fish were trained to accept formulated feed (P0) according to established protocols [29,30], and the formulation and proximate composition of the diets are presented in Table 1. A total of 360 healthy three-month-old fish (IBW: 85.57 ± 0.54 g) with uniform size and stable feeding response were selected for the trial. Fish were anesthetized with 20% MS-222 (Argent Chemical Laboratories, Redmond, WA, USA) for biometric measurements prior to random allocation into 18 circular tanks (1.6 m diameter × 0.9 m height), with 20 fish per tank. There were three replicate tanks for each experimental group (0 (P0), 400 (P1), 800 (P2), 1600 (P3), 3200 (P4), and 6400 mg/kg (P5)). Fish were fed twice daily (9:00 and 17:00) to apparent satiation, with feed ration adjusted to 3–5% of total biomass and 33–50% of daily water exchanged. Residual feed was collected and weighed after each feeding to calculate actual feed intake. During the acclimation and 8-week culture periods, the water quality was adjusted to maintain dissolved oxygen at 9.0 ± 0.2 mg/L, temperature at 19–24 °C, and pH at 7.8–8.1 [31].

### 2.3. Sample Collection

After the 8-week culture period, all experimental fish were weighed to record final body weights (FBWs). Six whole fish per experimental group (two fish from each of the 3 tanks) were collected and immediately flash-frozen in liquid nitrogen; then, they were stored at −20 °C for subsequent crude protein analysis. An additional six fish per experimental group (two fish from each of the 3 tanks) were anesthetized, rinsed superficially with 0.9% physiological saline, and dissected on ice using sterile surgical scissors. Brain, liver, and muscle tissues were rapidly excised, placed in aseptic centrifuge tubes, flash-frozen in liquid nitrogen, and stored at −80 °C for quantitative gene expression analysis [31,32].

### 2.4. Calculation of Growth Performance

The following growth parameters were calculated to evaluate nutritional utilization efficiency in Chinese perch:

Weight gain rate (WGR, %) = 100 × (final weight − initial weight)/initial weight(1)

Specific growth rate (SGR, %) = 100 × (Ln final weight − Ln initial weight)/day(2)

Feed rate (FR, %) = 100 × feed intake/((final weight + initial weight)/2 × day)(3)

Feed conversion ratio (FCR) = feed intake/weight gain(4)

Protein efficiency ratio (PER) = weight gain/protein intake(5)

Protein retention value (PRV) = protein gain/protein intake(6)

Growth performance data were modeled using broken-line regression analysis, with SGR and PRV as dependent variables against dietary choline supplementation concentrations [33].

### 2.5. Analysis of Crude Protein and Moisture Content

For proximate composition analysis, two fish per tank were sampled. Crude protein was measured by the Kjeldahl method, and the moisture content was measured by drying at 105 °C for 6 h following previous studies [34,35].

### 2.6. Gene Expression Quantification

Six fish per experimental group (two fish from each of the 3 tanks) were collected, and RNA was extracted independently from each fish. Total RNA was extracted from brain, hepatic, and muscular tissues of Chinese perch using the RNAiso Plus reagent kit (Takara, Dalian, China). RNA integrity and purity were assessed via 1.0% agarose gel electrophoresis and NanoDrop 2000 spectrophotometry (Thermo Scientific, Waltham, MA, USA), with acceptable samples defined by A_260_/A_280_ ratios of 1.8–2.2 [36]. Qualified RNA (200–1500 ng/μL) was reverse-transcribed into cDNA using the Evo M-MLV RT PreMix kit (Accura Biotechnology, Changsha, China) and stored at −80 °C [36].

Gene-specific primers (sequences listed in Table 2) were designed with Primer Premier 6.0 software and synthesized by Sangon Biotech Co., Ltd. (Shanghai, China). Quantitative real-time PCR (qRT-PCR) was performed on a Roche LightCycler 480^®^ system (Roche, Indianapolis, IN, USA) with the following 20 μL reaction mixture: 10 μL 2 × ChamQ Universal SYBR qPCR Master Mix, 0.4 μL forward primer, 0.4 μL reverse primer, 1 μL cDNA, and 8.2 μL DEPC-treated water (Novozyme, Nanjing, China). The qRT-PCR reaction program was as follows: 95 °C for 30 s, followed by 40 cycles of 95 °C for 5 s and 60 °C for 30 s; dissociation curve: 65–95 °C (0.5 °C increments, 5 s/step). All reactions were performed in triplicate. Relative gene expression was normalized to the ribosomal protein L13a reference gene (*rpl13a*) and calculated using the 2^−ΔΔCT^ method [37,38].

### 2.7. Statistical Analysis

All experimental data were expressed as mean ± standard error (SE) and visualized using GraphPad Prism 9.0 Software. Prior to analysis, data that deviated from the population mean were identified and excluded through a one-sample *t*-test (α = 0.05); meanwhile, the normality and homogeneity of variance were tested in SPSS 25.0 software. Statistical significance among groups was determined by one-way ANOVA and Duncan’s test. A probability level of *p* < 0.05 was considered statistically significant. Data marked with different lowercase letters represent statistically significant differences, while identical lowercase letters represent non-significant divergence (*p* > 0.05).

**Table 2:** Real-time primer sequence for Chinese perch.

Gene	Primer	Primer Sequence (5′-3′)	Tm (°C)	*E*-Value (%)
*gdh*	*gdh*-F *gdh*-R	GACGACGACCCCAACTTCT GACCCGCTTCCTCTTCTGC	58	94.3
*ampd*	*ampd*-F *ampd*-R	CATTTCCTTCCCGTGTT TCTGTCTGCGGAGTTGGT	58	103.6
*gk*	*gk*-F *gk*-R	AAGGTGGAGACCAAGAAC TGCCCTTGTCAATGTCC	58	96.9
*pk*	*pk*-F *pk*-R	CGCCCTCGCTGTCCTATTA TGCCGAAGTTGACCCTGTTG	57	99.9
*hsl*	*hsl*-F *hsl*-R	ACAAACGCCTGGGAATGGT TGTGGTCCGCCCTGAAGAA	58	99.6
*agrp*	*agrp*-F *agrp*-R	GTGCTGCTCTGCTGTTGG AGGTGTCACAGGGGTCGC	65	104
*npy*	*npy*-F *npy*-R	GGAAGGATACCCGGTGAAA TCTTGACTGTGGAATCGTG	52	107.2
*pomc*	*pomc*-F *pomc*-R	GGCTGAAGATGGTGTGTCTATG ACATGCAGAGGTGAATACAGTC	58	97.7
*cart*	*cart*-F *cart*-R	TCTGCACGAAGTGTTGGA GCACATCTTCCCGATACGA	56	95.3
*mafbx*	*mafbx*-F *mafbx*-R	AGCAGAACGTTCGTCCCATC GGGCCTGTTGATCTGGATGT	58	94.3
*murf1*	*murf1*-F *murf1*-R	TTTCGCCTGCCAGATCCATT TTGGGTCCAGTGTGCTCTTG	58	98.5
*mtor*	*mtor*-F *mtor*-R	GCATCAACGAGAGCACCA CGCTTCAAAATTCATAACCG	55	96.5
*s6k*	*s6k*-F *s6k*-R	CCTTCAAACCTTTCCTGCAATC ATTTAACTGGGCTGAGAGGTG	58	101.9
*pparα*	*pparα*-F *pparα*-R	GGGTGTGCTCAGACAAGGCT GTTGCGGTTCTTCTTTTGGAT	58	105.4
*pepck*	*pepck*-F *pepck*-R	CTGAGTTTGTGAAGAGAGCGG GTCCTTTGGGTCTGTGCGT	57	100.3
*eef2*	*eef2*-F *eef2*-R	TCTGCTGTTATCCCGCCT TCGCCATCACTCCTCCTCT	57.5	98.2
*lkb1*	*lkb1*-F *lkb1*-R	GACGGGGCATTTAAAATC GTGTTACTCCAGCAGACCAAA	57.5	98
*ampk*	*ampk*-F *ampk*-R	GGGATGCAAACCAAGATG ACAGACCCAGAGCGGAGC	57.5	101.7
*tor*	*tor*-F *tor*-R	GCATCAACGAGAGCACCA CGCTTCAAAATTCATAACGC	57.5	96.5
*rpl13a*	*rpl13a*-F *rpl13a*-R	CACCCTATGACAAGAGGAAGC TGTGCCAGACGCCCAAG	59	102.9

Note: Tm: melting temperature.

## 3. Results

### 3.1. Growth Performance

The effects of dietary choline supplementation on the growth performance of Chinese perch are presented in Table 3. For WGR and SGR, only the P2 and P3 groups showed significant increasing trends compared to the P0 group (*p* < 0.05). Only the P2 group showed a significantly higher FR than that of the P0 group (*p* < 0.05). Compared to the P0 group, PER and PRV significantly increased in the P1, P2, P3, and P4 groups, but FCR significantly decreased in the P2, P3, and P4 groups (*p* < 0.05). However, the P5 group showed markedly lower SGR, PER, and PRV than those of the P0 group (*p* < 0.05).

**Figure 1:**
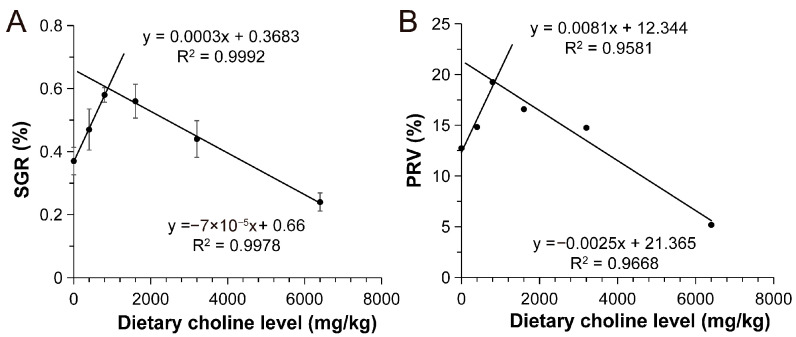
The relationship of SGR (**A**) and PRV (**B**) with dietary choline supplementation in different choline-supplemented groups and the control group.

Linear regression analyses revealed biphasic relationships between the choline supplementation levels and the growth indices (Figure 1). SGR displayed a strong positive correlation (R^2^ = 1.00) with choline supplemented below 800 mg/kg, followed by a negative correlation (R^2^ = 1.00) with choline supplementation at higher concentrations (1600–6400 mg/kg) (Figure 1A). Similarly, PRV exhibited a positive correlation (R^2^ = 0.96) with choline supplementation (0–800 mg/kg) and a negative correlation (R^2^ = 0.97) with choline supplementation at elevated concentrations (1600–6400 mg/kg) (Figure 1B). Model fitting identified optimal choline supplementation levels at 788.38 mg/kg for SGR and 851.04 mg/kg for PRV in Chinese perch.

### 3.2. Crude Protein Content

**Figure 2:**
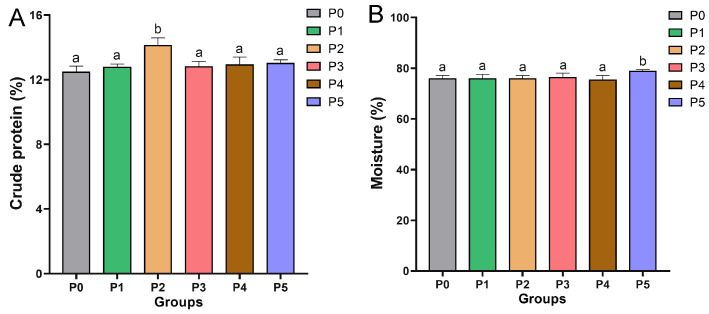
The crude protein (**A**) and moisture content (**B**) in different choline-supplemented groups and the control group. Different lowercase letters indicate significant differences between groups (*p* < 0.05), while the same lowercase letters indicate no significant differences (*p* > 0.05).

The effects of dietary choline supplementation on the crude protein and moisture content in Chinese perch are illustrated in Figure 2. The crude protein contents of fish in the P0, P1, P2, P3, P4, and P5 groups were 12.50 ± 0.20%, 12.81 ± 0.10%, 14.15 ± 0.25%, 12.83 ± 0.18%, 12.95 ± 0.26%, and 13.04 ± 0.11% of wet weight, respectively. Only the P2 group showed a significantly higher crude protein content than the P0 group (*p* < 0.05), while no significant differences were observed in the crude protein contents among the other five groups (*p* > 0.05). The moisture contents of fish in the P0, P1, P2, P3, and P4 groups were 76.10 ± 0.97%, 76.10 ± 1.46%, 76.10 ± 0.97%, 76.58 ± 1.47%, and 75.61 ± 1.46%, respectively, and there were no significant differences among the five groups (*p* > 0.05). However, the moisture content of fish in the P5 group (79.01 ± 0.48%) was significantly higher than that in the P0 group (*p* < 0.05).

### 3.3. Expression Level of Feeding-Related Genes

**Figure 3:**
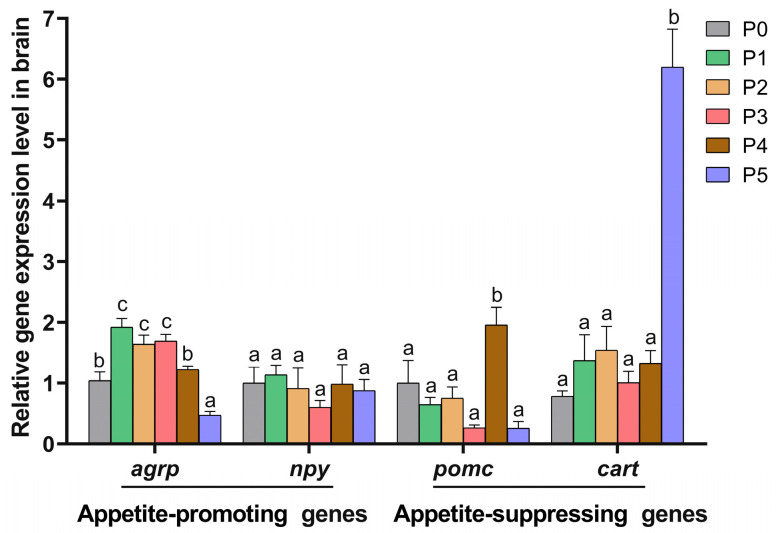
Feeding-related gene expression levels in the brain in different choline-supplemented groups and the control group. Different lowercase letters indicate significant differences between groups (*p* < 0.05), while the same lowercase letters indicate no significant differences (*p* > 0.05).

The effects of dietary choline supplementation on feeding-related gene expression in the brain of Chinese perch are presented in Figure 3. Compared to the P0 group, the expression levels of the orexigenic gene *agrp* in the P1, P2, and P3 groups were significantly higher (*p* < 0.05), while there was a significant decrease in the P5 group (*p* < 0.05) and no significant change in the P4 group (*p* > 0.05). No significant difference was found in the expression level of the orexigenic gene *npy* among all groups (*p* > 0.05).

Compared to the P0 group, only the expression level of the anorexigenic gene *pomc* in the P4 group significantly increased, but there were no apparent changes in the other groups (*p* > 0.05). The expression levels of the anorexigenic gene *cart* had no significant change in the P1, P2, P3, and P4 groups compared to the P0 group (*p* > 0.05), while a significant increase in the *cart* gene expression level was found in the P5 group (*p* < 0.05).

### 3.4. Expression Level of Protein Metabolism-Related Genes

**Figure 4:**
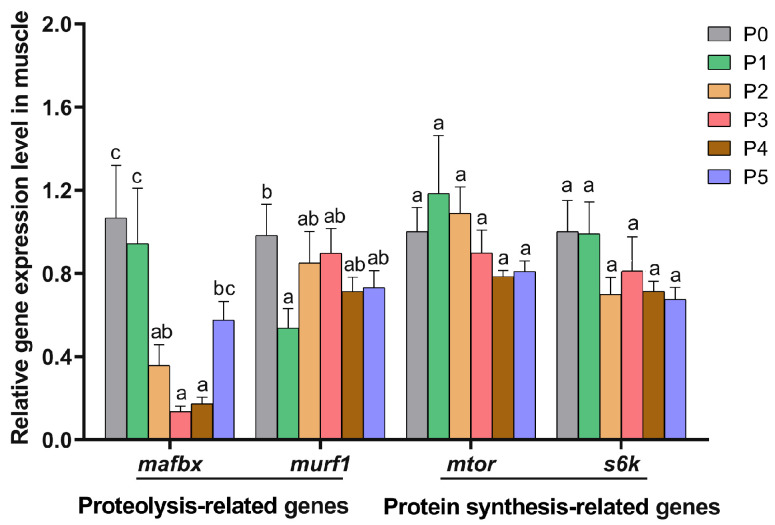
Protein metabolism-related gene expression levels in the muscle in different choline-supplemented groups and the control group. Different lowercase letters indicate significant differences between groups (*p* < 0.05), while the same lowercase letters indicate no significant differences (*p* > 0.05).

**Figure 5:**
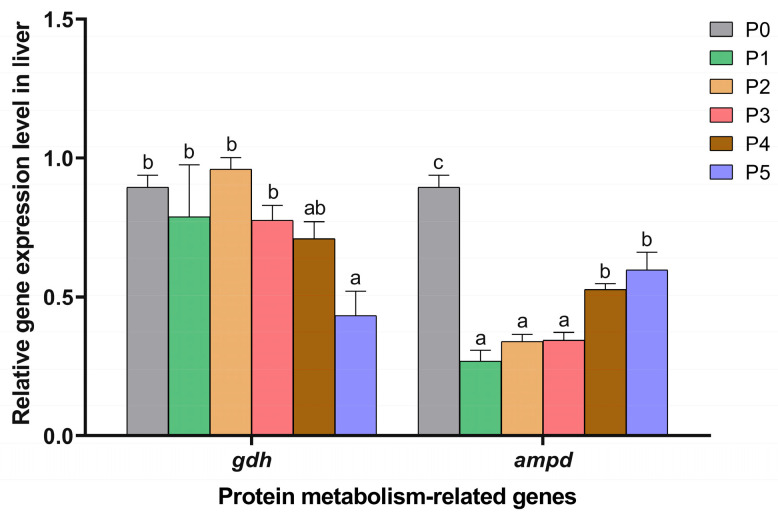
Protein metabolism-related gene expression levels in the liver in different choline-supplemented groups and the control group. Different lowercase letters indicate significant differences between groups (*p* < 0.05), while the same lowercase letters indicate no significant differences (*p* > 0.05).

The effects of dietary choline supplementation on protein metabolism-related gene expression in the muscle and liver of Chinese perch are given in Figure 4 and Figure 5, respectively. The relative expression levels of the proteolytic gene *mafbx* were significantly down-regulated in the P2, P3, and P4 groups compared to the P0 group (*p* < 0.05), with no significant changes in the P1 and P5 groups (*p* > 0.05). Compared to the P0 group, only the P1 group exhibited a significantly lower *murf1* gene expression level (*p* < 0.05). There were no significant differences in the expression levels of protein synthesis-related genes (*mtor* and *s6k*) among all groups (*p* > 0.05).

As shown in Figure 5, only the expression level of the deamination gene *gdh* in the P5 group was significantly depressed compared to that in the P0 group (*p* < 0.05), while it showed no significant changes among the P1, P2, P3, P4, and P0 groups (*p* > 0.05). However, the P1, P2, P3, P4, and P5 groups exhibited significantly lower *ampd* gene expression levels than the P0 group (*p* < 0.05).

### 3.5. Expression Level of Glucose and Lipid Metabolism-Related Genes

**Figure 6:**
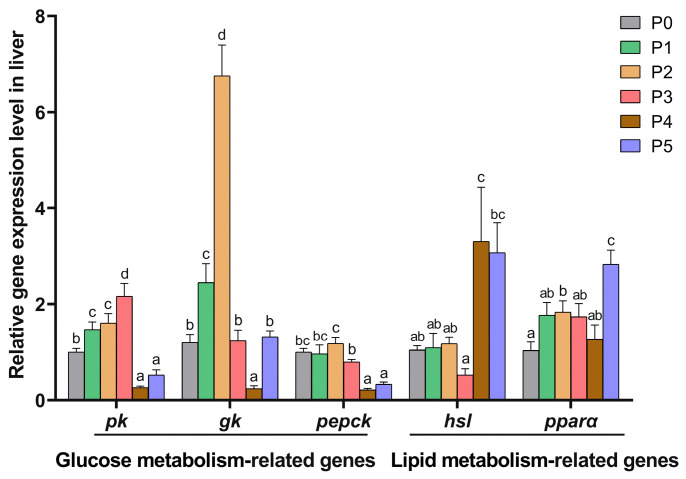
Glucose and lipid metabolism-related gene expression levels in the liver in different choline-supplemented groups and the control group, respectively. Different lowercase letters indicate significant differences between groups (*p* < 0.05), while the same lowercase letters indicate no significant differences (*p* > 0.05).

The effects of choline supplementation on the expression levels of the hepatic glucose and lipid metabolism-related genes of Chinese perch are displayed in Figure 6. Compared to the P0 group, the expression levels of the hepatic glycolytic gene *pk* were significantly elevated in the P1, P2, and P3 groups (*p* < 0.05), while they were significantly decreased in the P4 and P5 groups (*p* < 0.05). The expression levels of the hepatic glycolytic *gk* gene increased significantly in the P1 and P2 groups but decreased in the P4 group (*p* < 0.05) compared to the P0 group. The expression levels of the gluconeogenic gene *pepck* were significantly down-regulated in the P4 and P5 groups (*p* < 0.05). Compared to the P0 group, the expression levels of the lipolytic gene *hsl* increased significantly in the P4 group (*p* < 0.05), while the expression levels of the lipolytic gene *pparα* increased markedly in the P2 and P5 groups (*p* < 0.05).

### 3.6. Expression Level of Energy Metabolism-Related Genes

The AMP-activated protein kinase (AMPK) and target of rapamycin (TOR) pathways are critical signaling cascades that regulate cellular energy homeostasis.

**Figure 7:**
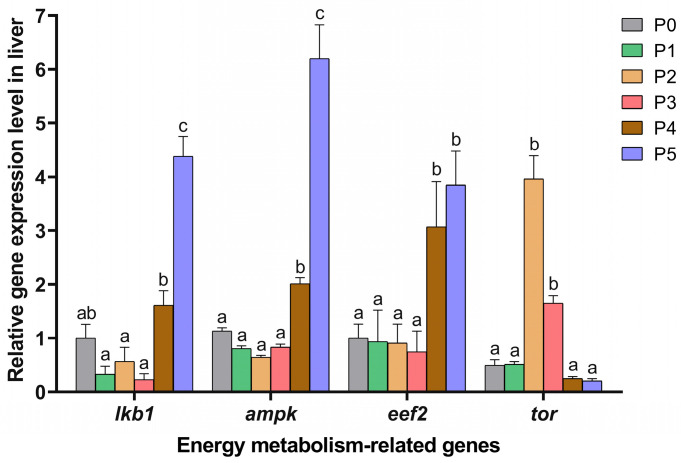
Energy metabolism-related gene expression levels in the liver in different choline-supplemented groups and the control group. Different lowercase letters indicate significant differences between groups (*p* < 0.05), while the same lowercase letters indicate no significant differences (*p* > 0.05).

As shown in Figure 7, no significant differences were observed in the expression levels of AMPK pathway-related genes (*lkb1*, *ampk*, and *eef2*) among the P1, P2, P3, and P0 groups (*p* > 0.05). However, significant increases in *ampk* and *eef2* gene expression levels were seen in the P4 and P5 groups compared to the P0 group (*p* < 0.05). In addition, the expression level of the *lkb1* gene was higher in the P5 group than in the P0 group (*p* < 0.05). Notably, the expression level of the *tor* gene in the P2 and P3 groups was significantly higher than those in the other four groups (*p* < 0.05).

## 4. Discussion

### 4.1. Effects of Dietary Choline Supplementation on the Growth Performance and Feeding of Chinese Perch

The present study demonstrated a dose-dependent relationship between choline supplementation and growth performance in Chinese perch. Compared with the control group, dietary supplementation with 400, 800, 1600, and 3200 mg/kg of choline supplementation significantly increased the PER and PRV of Chinese perch (*p* < 0.05). Choline supplementation at 800 and 1600 mg/kg also increased SGR and WGR but significantly reduced FCR (*p* < 0.05). In addition, 800 mg/kg of choline supplementation significantly improved the FR of Chinese perch (*p* < 0.05). These findings are consistent with a previous study on grass carp, which reported that 385–1795 mg/kg of choline supplementation improved the feed intake (FI) of grass carp, which had a strong positive correlation with WGR and feed efficiency [13]. Similarly, Wu et al. observed that 607–1820 mg/kg of choline supplementation improved the FBW, SGR, PRV, FI, and intestinal protease activity of common carp (*Cyprinus carpio*) [39]. Also, 2024 mg/kg of choline supplementation enhanced the SGR and protein productive value of gibel carp (*Carassius auratus gibelio*) [40]. Many studies have indicated that choline could not only promote the feeding behavior of fish by modulating the expression of orexigenic genes but also improve their digestive capacity by increasing the secretion of intestinal enzymes [41,42,43], which could be part of the reason for the improvements in the growth performance and feeding of Chinese perch in this study. Moreover, the positive effects of choline on fish growth could also be attributed to its other roles in enhancing intestinal digestive enzyme activity, improving glucose and lipid metabolism, and promoting protein synthesis [21,22,23,43].

Choline-induced improvements in the feed intake of fish are associated with the modulation of appetite and energy metabolism [42]. Key appetite-regulating neuropeptides include orexigenic factors such as neuropeptide Y (NPY) and agouti-related peptide (AgRP) and anorexigenic factors like proopiomelanocortin (POMC) and cocaine- and amphetamine-regulated transcript (CART) [44,45]. In this study, choline supplementation (800 mg/kg) significantly increased FR (*p* < 0.05). In addition, 400–1600 mg/kg of choline supplementation was found to up-regulate *agrp* gene expression in the brain (*p* < 0.05), indicating that choline could enhance the feeding behavior of Chinese perch by stimulating *agrp* gene expression, thereby improving their growth performance. Broken-line regression analysis revealed a parabolic relationship between choline supplementation and the growth performance of Chinese perch. Both SGR and PRV initially increased and subsequently decreased with rising choline supplementation levels. The maximal SGR was achieved at 788.38 mg/kg of choline supplementation, while peak PRV occurred at 851.04 mg/kg of choline supplementation, which suggested that the optimal choline supplementation level is 788.38 mg/kg based on SGR and 851.04 mg/kg based on PRV. Comparative analysis with previous studies demonstrated the species-specific variability in choline requirements. The optimal choline supplementation level for Chinese perch exceeds those for Nile tilapia (400 mg/kg) and yellow perch (*Perca flavescens*, 598–634 mg/kg) [21,46] but remains lower than the choline supplementation levels for orange-spotted grouper (1094 mg/kg), grass carp (1137 mg/kg), blunt snout bream (*Megalobrama amblycephala*, 1198 mg/kg), gibel carp (2500 mg/kg), and yellowtail kingfish (*Seriola lalandi*, 3000 mg/kg) [13,16,17,40,47]. These discrepancies are likely due to many factors, including environmental conditions, diet composition, fish species, and developmental stages [13,17,24,40,47].

It has been reported that when the added choline exceeded the optimal level, the growth performance of fish could be reduced. For example, 4029 mg/kg of choline supplementation decreased FW and SGR in the orange-spotted grouper [24], while 20,000 mg/kg of choline supplementation decreased the growth performance and survival rate of the hybrid grouper [19]. Similar negative effects of high-level dietary choline supplementation have been illustrated in Senegalese sole (*Solea senegalensis*) and grass carp [13,48]. In this study, 3200 mg/kg of choline supplementation significantly up-regulated the anorexigenic gene *pomc* (*p* < 0.05). Also, 6400 mg/kg of choline supplementation increased the expression of the anorexigenic gene *cart* (*p* < 0.05) while suppressing the expression of the orexigenic gene *agrp* (*p* < 0.05). Concurrently, 6400 mg/kg of choline supplementation significantly decreased WGR, SGR, PER, and PRV while increasing FCR (*p* < 0.05). These results suggested that excessive choline supplementation (3200 and 6400 mg/kg) disrupted appetite regulation and nutrient assimilation, ultimately decreasing the growth performance of Chinese perch.

### 4.2. Effects of Dietary Choline Supplementation on the Protein and Energy Metabolism Gene Expressions of Chinese Perch

**Figure 8:**
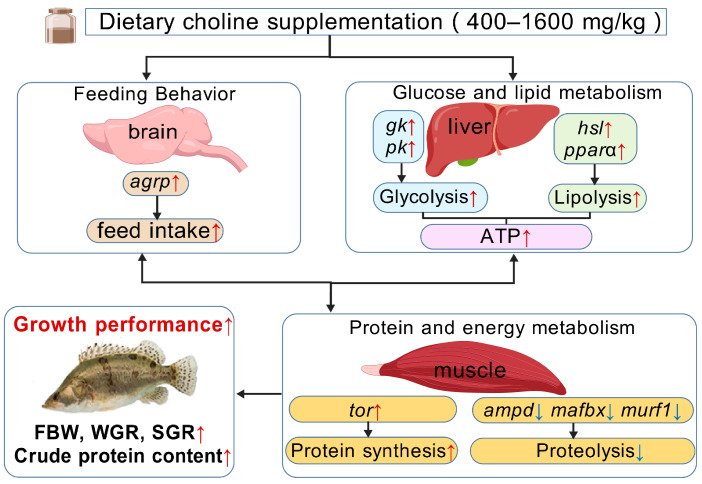
The mechanism of dietary choline supplementation promoting the growth performance of Chinese perch. Red arrows indicate an increase in the parameters or expression levels of related genes in the pathway. Blue arrows represent a decrease in the expression levels of related genes in the pathway.

Muscle ring finger protein-1 (MuRF-1) and muscle atrophy F-box protein (MAFbx), members of the ubiquitin ligase E3 family, are key biomarkers of proteolysis [49]. In this study, dietary choline supplementation at 400 mg/kg significantly reduced the expression level of the muscular proteolysis gene *murf1* (*p* < 0.05). Choline supplementation at 800–3200 mg/kg also decreased the expression level of the muscular proteolysis gene *mafbx* (*p* < 0.05). Furthermore, 400–6400 mg/kg of choline supplementation significantly reduced the expression level of the AMP deaminase gene (*ampd*) in the muscle (*p* < 0.05). These results were correlated with elevated PRV and PER in the choline-supplemented groups (P1, P2, P3, and P4). Comparable results were reported in a study of common carp, which confirmed that 607–1820 mg/kg of choline supplementation up-regulated the expression of protein synthesis-related genes (*tor* and *4ebp2*) and improved PRV and muscle protein content [39]. Collectively, our results suggested that 400–3200 mg/kg of choline supplementation reduced protein degradation and promoted amino acid utilization, thereby improving dietary protein utilization of Chinese perch (Figure 8).

The metabolic regulatory role of choline is mediated through its provision of active methyl groups for energy metabolism [9]. Methylation facilitates phospholipid synthesis, carnitine production, and methionine regeneration, so it promotes energy generation from carbohydrate and lipid catabolism and inhibits protein catabolism [19,42,50]. Eukaryotic elongation factor 2 (eEF2), a critical regulator of protein synthesis, is modulated by the AMP-activated protein kinase (AMPK) pathway [6]. When cellular energy supply is insufficient, the liver kinase B1 (LKB1) phosphorylates and activates AMPK, leading to inhibitory phosphorylation of eEF2 and subsequent suppression of protein synthesis [51,52]. Conversely, the target of rapamycin (TOR) pathway drives protein synthesis during energy sufficiency [53,54]. When cellular energy is sufficiently available, the TOR pathway is activated, thereby promoting cell growth [53,54].

In this study, 800 and 1600 mg/kg of choline supplementation significantly increased the expression level of the *tor* gene (*p* < 0.05) in the liver, concomitant with the elevated WGR, SGR, PER, and PRV (*p* < 0.05) in the P2 and P3 groups (Figure 8). A similar up-regulation of *tor* gene expression and growth enhancement were also observed in grass carp fed with 197–770 mg/kg of choline supplementation [13]. Dietary supplementation with choline (800–2000 mg/kg) increased intestinal *tor* and *s6k1* gene expression in grass carp [25]. Baldissera et al. further demonstrated that 800–1200 mg/kg of choline supplementation enhanced hepatic ATP production to support the rapid growth of Nile tilapia [21]. However, excessive choline supplementation (6400 mg/kg) significantly up-regulated the gene expression of energy stress markers (*lkb1*, *ampk*, and *eef2*) (*p* < 0.05), which may be due to the imbalance of energy metabolism in fish caused by the high levels of choline supplementation. These results indicated that 6400 mg/kg of choline supplementation activated the AMPK pathway, inhibited protein synthesis, promoted protein catabolism, and ultimately reduced the growth performance of fish [1,19], which corresponded to the lowest WGR, SGR, PER, and PRV observed in the P5 group.

### 4.3. Effects of Dietary Choline Supplementation on the Glucose and Lipid Metabolism Gene Expressions of Chinese Perch

Carbohydrates and lipids are crucial energy substrates in fish, and glycolysis and the tricarboxylic acid (TCA) cycle facilitate aerobic glucose oxidation to meet metabolic demands [31]. An enhanced catabolism of these substrates ensures sufficient energy availability, thereby promoting protein synthesis, deposition, and the growth of fish [31]. As key enzymes in glycolysis and gluconeogenesis, glucokinase (GK), pyruvate kinase (PK), and phosphoenolpyruvate carboxykinase (PEPCK) are essential to these processes [20,55]. Studies have demonstrated that the positive impact of choline supplementation on the growth performance of fish may be related to its regulatory role in glucose and lipid metabolism [56]. Geng et al. found that 2590 mg/kg of choline supplementation elevated *pk* and *pepck* gene expression levels in the liver and muscle of largemouth bass (*Micropterus salmoides*) [57]. Baldissera et al. also reported that 800–1200 mg/kg choline supplementation increased the hepatic PK activity and ATP production in Nile tilapia [21]. In this study, 400 and 800 mg/kg of choline supplementation significantly promoted the expression levels of the hepatic glycolytic gene (*pk* and *gk*) (*p* < 0.05), and 1600 mg/kg of choline supplementation also enhanced *pk* gene expression (*p* < 0.05) (Figure 8), suggesting that glucose catabolism was enhanced after choline supplementation. However, 3200 and 6400 mg/kg of choline supplementation suppressed glycolytic gene (*pk* and *gk*) expression in the liver of Chinese perch (*p* < 0.05), suggesting inhibitory effects of high-level choline supplementation on carbohydrate utilization of fish.

Hormone-sensitive lipase (HSL) and the peroxisome proliferator-activated receptor alpha (PPARα) pathway are pivotal regulators of lipid catabolism [58,59]. Choline supplementation in fish feed was reported to enhance lipid metabolism by up-regulating lipolytic gene expression (*hsl* and *pparα*), stimulating lipoprotein lipase (LPL) and hepatic lipase (HL) activities, and promoting carnitine and lipoprotein syntheses [23,26,43,49]. In this study, 800 and 6400 mg/kg of choline supplementation increased the *pparα* gene expression level. Also, 3200 mg/kg of choline supplementation induced the *hsl* gene expression level (Figure 8). Comparable findings in yellow catfish showed that 1658.4 mg/kg of choline supplementation up-regulated lipolysis gene expression (*hsl*, *cpt-1α*, and *echs1*), while it down-regulated lipogenic gene expression (*pparγ*, *accα*, and *fas*) [26].

In summary, as shown in Figure 8, this study found that moderate dietary choline supplementation (400–1600 mg/kg) not only up-regulated the orexigenic gene expressions to promote fish feeding behavior but also enhanced protein synthesis by increasing the expression levels of glycolysis- and lipolysis-related genes and suppressing those of proteolysis-related genes, resulting in an enhanced growth performance of Chinese perch.

## 5. Conclusions

The present study demonstrated that dietary choline supplementation at 400–1600 mg/kg improved WGR, SGR, PER, and PRV while reducing FCR in Chinese perch. However, choline supplementation at 6400 mg/kg reduced the growth performance of Chinese perch. Moderate dietary choline supplementation up-regulated glycolysis- and lipolysis-related genes and down-regulated proteolysis-related genes, suggesting that choline supplementation could enhance glucose and lipid catabolism, reduce protein degradation, and promote protein synthesis and deposition, resulting in improved energy availability, growth performance, and feed efficiency in Chinese perch. Broken-line analysis indicated that the optimal choline supplementation level for Chinese perch is 788.38 mg/kg based on SGR and 851.04 mg/kg based on PRV.

## Figures and Tables

**Table 1:** Feed formulation for Chinese perch.

Ingredients (%)	P0	P1	P2	P3	P4	P5
Fish meal ^1^	47.00	47.00	47.00	47.00	47.00	47.00
Casein	20.00	20.00	20.00	20.00	20.00	20.00
Starch	8.00	8.00	8.00	8.00	8.00	8.00
Fish oil	4.00	4.00	4.00	4.00	4.00	4.00
Soybean oil	4.00	4.00	4.00	4.00	4.00	4.00
Vitamin premix ^2^	2.00	2.00	2.00	2.00	2.00	2.00
Mineral premix ^3^	2.00	2.00	2.00	2.00	2.00	2.00
Microcrystalline cellulose	8.00	7.92	7.84	7.68	7.36	6.72
CaHPO_4_	2.00	2.00	2.00	2.00	2.00	2.00
Trialgin ^4^	3.00	3.00	3.00	3.00	3.00	3.00
Choline chloride (50%)	0.00	0.08	0.16	0.32	0.64	1.28
Proximate composition						
Crude protein	46.06	46.06	46.06	46.06	46.06	46.06
Crude lipid	11.88	11.88	11.88	11.88	11.88	11.88
Carbohydrate	7.98	7.98	7.98	7.98	7.98	7.98

^1^ per kg of fish meal had 3000 mg/kg choline; thus, the level of choline in the control diet P0 was 1410 mg/kg. ^2^ Vitamin premix (per kg of diet): inositol 600 mg, vitamin A 40 mg, vitamin D_3_ 0.06 mg, vitamin E 200 mg, vitamin K_3_ 10 mg, vitamin B_1_ (thiamine) 15 mg, vitamin B_2_ (riboflavin) 25 mg, vitamin B_6_ 20 mg, pantothenic acid 50 mg, vitamin B_3_ (nicotinic acid) 200 mg, biotin 3.2 mg, vitamin B_12_ 0.1 mg, folic acid 10 mg, vitamin C 210 mg. ^3^ Mineral premix (per kg of diet): CaHPO_4_ 94.9 g, KCl 5.45 g, MgSO_4_ 4 g, NaCl 3.8 mg, CuSO_4_ 25 mg, FeSO_4_ 407 mg, ZnSO_4_ 198 mg, MnSO_4_ 36 mg, Na_2_SeO_3_ 1.8 mg, KI 1.4 mg, Na_2_MoO_4_ 0.34 mg, CoSO_4_ 0.09 mg, KF 0.8 mg. ^4^ Trialgin (per kg of diet): kelp 330 g, *Gracilaria* 330 g, laver 270 g, *Spirulina* 10 g, *Azolla* 30 g, wakame 30 g.

**Table 3:** The growth performance parameters of Chinese perch.

Parameter	P0	P1	P2	P3	P4	P5
IBW (g)	86.18 ± 2.46 ^a^	82.47 ± 1.58 ^a^	85.45 ± 2.44 ^a^	83.18 ± 2.38 ^a^	89.25 ± 2.35 ^a^	86.91 ± 1.02 ^a^
FBW (g)	111.60 ± 2.38 ^ab^	114.38 ± 3.09 ^abc^	127.79 ± 5.41 ^c^	122.24 ± 4.59 ^bc^	121.08 ± 5.88 ^bc^	102.57 ± 1.87 ^a^
WGR (%)	29.56 ± 3.81 ^ab^	38.72 ± 3.32 ^bcd^	49.44 ± 2.35 ^d^	47.10 ±5.67 ^cd^	35.41 ± 5.01 ^bc^	18.02 ± 2.16 ^a^
SGR (%)	0.37 ± 0.04 ^b^	0.47 ± 0.03 ^bcd^	0.58 ± 0.02 ^d^	0.56 ± 0.06 ^cd^	0.44 ± 0.05 ^bc^	0.24 ± 0.03 ^a^
FR (%)	1.14 ± 0.01 ^ab^	1.28 ± 0.01 ^abc^	1.49 ± 0.09 ^d^	1.43 ± 0.15 ^bc^	1.26 ± 0.16 ^abc^	1.04 ± 0.06 ^a^
FCR	2.74 ± 0.05 ^d^	2.58 ± 0.03 ^d^	1.82 ± 0.05 ^a^	2.10 ± 0.01 ^b^	2.37 ± 0.06 ^c^	3.57 ± 0.19 ^e^
PER (%)	75.95 ± 1.41 ^b^	88.34 ± 1.04 ^c^	114.79 ± 3.06 ^e^	98.88 ± 0.46 ^d^	87.94 ± 2.16 ^c^	30.89 ± 1.60 ^a^
PRV (%)	12.74 ± 0.24 ^b^	14.81 ± 0.17 ^c^	19.25 ± 0.51 ^e^	16.58 ± 0.08 ^d^	14.75 ± 0.36 ^c^	5.18 ± 0.27 ^a^

Note: Different lowercase letters indicate significant differences between groups (*p* < 0.05), while the same lowercase letters indicate no significant differences (*p* > 0.05).

## Data Availability

The original contributions presented in this study are included in the article. Further inquiries can be directed to the corresponding authors.
